# Fingerprints of sp^1^ Hybridized C in the Near-Edge X-ray Absorption Spectra of Surface-Grown Materials

**DOI:** 10.3390/ma11122556

**Published:** 2018-12-15

**Authors:** Guido Fratesi, Simona Achilli, Nicola Manini, Giovanni Onida, Anu Baby, Abhilash Ravikumar, Aldo Ugolotti, Gian Paolo Brivio, Alberto Milani, Carlo Spartaco Casari

**Affiliations:** 1 ETSF and Dipartimento di Fisica, Università degli Studi di Milano, Via Celoria, 16, I-20133 Milano, Italy; simona.achilli@unimi.it (S.A.); nicola.manini@fisica.unimi.it (N.M.); Giovanni.Onida@mi.infn.it (G.O.); 2 Dipartimento di Scienza dei Materiali, Università di Milano-Bicocca, Via Cozzi, 55, 20125 Milano, Italy; a.baby@campus.unimib.it (A.B.); a.ravikumar@campus.unimib.it (A.R.); a.ugolotti@campus.unimib.it (A.U.); gianpaolo.brivio@unimib.it (G.P.B.); 3 Department of Energy, Politecnico di Milano via Ponzio 34/3, I-20133 Milano, Italy; alberto.milani@polimi.it (A.M.); carlo.casari@polimi.it (C.S.C.)

**Keywords:** carbynes, near edge X-ray absorption spectroscopy, self-assembly, density functional theory, C 1s absorption, on-surface chemistry

## Abstract

Carbon structures comprising sp${}^{1}$ chains (e.g., polyynes or cumulenes) can be synthesized by exploiting on-surface chemistry and molecular self-assembly of organic precursors, opening to the use of the full experimental and theoretical surface-science toolbox for their characterization. In particular, polarized near-edge X-ray absorption fine structure (NEXAFS) can be used to determine molecular adsorption angles and is here also suggested as a probe to discriminate sp${}^{1}$/sp${}^{2}$ character in the structures. We present an ab initio study of the polarized NEXAFS spectrum of model and real sp${}^{1}$/sp${}^{2}$ materials. Calculations are performed within density functional theory with plane waves and pseudopotentials, and spectra are computed by core-excited C potentials. We evaluate the dichroism in the spectrum for ideal carbynes and highlight the main differences relative to typical sp${}^{2}$ systems. We then consider a mixed polymer alternating sp${}^{1}$ C${}_{4}$ units with sp${}^{2}$ biphenyl groups, recently synthesized on Au(111), as well as other linear structures and two-dimensional networks, pointing out a spectral line shape specifically due to the the presence of linear C chains. Our study suggests that the measurements of polarized NEXAFS spectra could be used to distinctly fingerprint the presence of sp${}^{1}$ hybridization in surface-grown C structures.

## 1. Introduction

The groundbreaking results achieved on graphene in the last decade have stimulated the interest in novel carbon allotropes and nanostructures [1,2,3,4,5]. Amongst all, the infinite carbon monoatomic wire, i.e., carbyne, represents a carbon allotrope based on sp${}^{1}$ hybridization, which joins graphite and diamond based on sp${}^{2}$ and sp${}^{3}$ hybridization, respectively [6]. Carbyne is the ideal ultimate one-dimensional (1D) carbon nanostructure and related systems have been widely investigated in the late 1980s [7,8]. A renewed attention has grown in more recent years due to the outstanding properties predicted by a number of theoretical calculations [8]. The occurrence of carbyne has been considered improbable or even impossible by the scientific community due to stability issues [9]. However recent examples have shown the feasibility of long and stable sp${}^{1}$-carbon wires either stabilized by suitable terminations [10], or inserted in the core of carbon nanotubes [11] or by rotaxanes chemistry [12,13,14,15,16]. These achievements have also renewed the interest in mixed sp${}^{1}$-sp${}^{2}$ structures, such as the two-dimensional (2D) crystals graphyne and graphdiyne, showing the potential to compete with graphene [17,18,19,20,21].

In the last 20 years many different techniques were developed for synthesizing sp${}^{1}$-carbon nanostructures. The main strategies can be divided into physical-based and chemical-based. Physical methods are usually based on the clustering from a carbon vapour or plasma in out-of-equilibrium conditions and span from laser ablation to arch discharge, both in gases and in liquids [22,23,24,25]. Chemical-based techniques exploit rational synthesis methods such as polycondensation reactions, dehydrogenation of polymers and dehalogenation reactions to achieve fine control on wire length and termination [26,27,28,29]. A recent strategy which somehow merges chemical and physical methods relies on the on-surface synthesis of sp${}^{1}$/sp${}^{2}$ carbon structures by dehalogenation and homocoupling reaction of suitably tailored molecular precursors. In 2016, Wei Xu et al. evaporated Br-terminated molecular precursors containing sp carbon on the Au(111) surface in ultra-high vacuum (UHV) conditions [30]. By means of scanning tunneling microscopy (STM) they demonstrated the surface-mediated dehalogenation of the precursor and the formation of long sp${}^{1}$-sp${}^{2}$ carbon wires and a new 2D sp${}^{1}$-sp${}^{2}$ system belonging to the family of graphdiynes. On-surface synthesis has been performed also with Cl-functionalized precursors on Cu(111) and even for the synthesis of cumulene-like systems (i.e., sp${}^{1}$-carbon wires with all equal double bonds) [30,31,32]. These works reported the images of single sp${}^{1}$-carbon wires with resolution at molecular-bond level, obtained by the combined use of STM and atomic force microscopy (AFM).

Though providing precious structural information on the investigated systems, STM and AFM cannot provide sufficient insight into the nature of the chemical bond and the electronic properties, thus requiring complementary techniques such as photoemission and absorption spectroscopy. In this context, examples of the use of common surface-science techniques to investigate sp${}^{1}$-carbon systems are rare and further work is needed to standardize these techniques for the identification and detailed study of sp${}^{1}$-carbon structures [33,34]. Specifically, near-edge X-ray absorption spectroscopy (NEXAFS) [35,36] is a powerful tool widely applied to provide information on the empty electronic states and on molecular orientations of organic sp${}^{2}$ systems at surfaces, by exploiting the relation of the spectral dependence on photon polarization (dichroism) with the experimental geometry for linearly polarized light. While various contributions in the literature consider the use of X-ray photoemission (XPS) and NEXAFS tools for discriminating carbon hybridization (see, e.g., [33,34,37,38,39,40]) none of them, to our knowledge, exploits such dependence. In this context, the applicability of NEXAFS to sp${}^{1}$ adsorbed cases deserves a detailed investigation.

On the theoretical standpoint, Density Functional Theory (DFT) [41,42] seems the best compromise between accuracy and computational cost for computing the core-level spectral features of moderately large systems, as those necessary to simulate surface-deposited molecules or polymers. The large separation between core and valence energies facilitates the description of the excited system by approximately evaluating the outer electronic structure in the presence of a core-level excitation, even though new implementations are becoming available that include many-body correlation effects [43]. In this respect, the half-core-hole approximation [44,45] appears to be quite adequate for molecular and adsorbed systems [46], and was validated for a variety of carbon-based systems [47,48,49,50,51]. The dichroism in the NEXAFS spectra is well reproduced by the simulations allowing for a detailed identification of adsorption orientations [52] and molecular-orbital symmetry [53].

In this work we report ab initio numerical simulations of the electronic and spectral (NEXAFS) properties of model configurations and realistic ones, involving linear sp${}^{1}$ carbon chains. We first compare the spectral characteristics of metallic and semiconducting carbynes, and identify the dichroism in the NEXAFS spectra for model sp${}^{1}$ and sp${}^{2}$ systems. We then focus on a paradigmatic mixed sp${}^{1}$/sp${}^{2}$ C polymer that was recently synthesized on Au(111). Our simulations here include the influence of the substrate on the electronic properties. The emergence of sp${}^{1}$-related features in the spectra is pointed out and proposed for experiments identifying sp${}^{1}$ character. Other structural models referring to intermediate stages of this polymer growth, as well as to 2D networks, are then presented.

## 2. Results

### 2.1. Model 1D Systems

**Figure 1:**
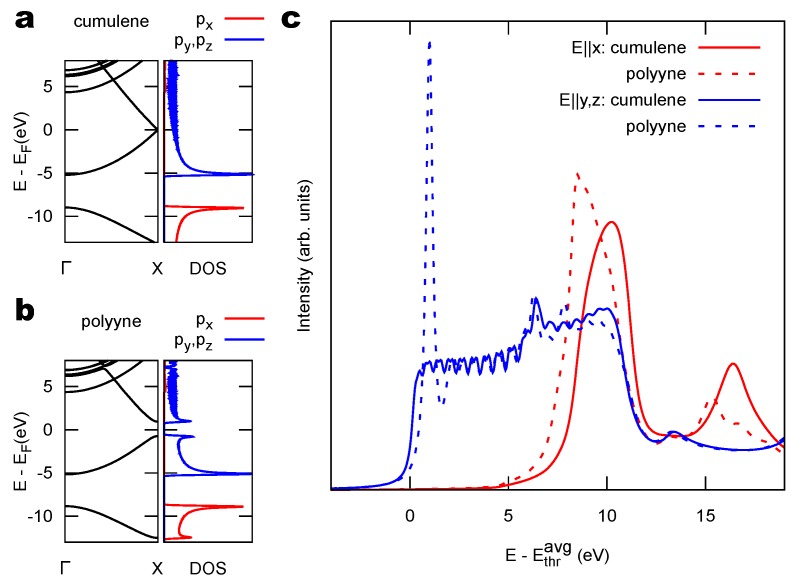
(**a**,**b**) Band structure (left) and corresponding density of states (right) for infinite 1D carbon chains of the cumulene family (=C=C=, BLA=0) and polyyne one (-C ≡ C-, BLA = 0.172 Å) [54], respectively. The DOS is projected over C 2p atomic orbitals, with *x* along the chain axis and y,z orthogonal to it. (**c**) Simulated near-edge X-ray absorption fine structure (NEXAFS) spectrum for different photon polarizations. Energies are reported with reference to the average C 1s ionization potential $E_{\mathrm{thr}}^{\mathrm{avg}}$. The wiggles that are especially evident at low energy are an artifact induced by the use of periodically repeated unit cells (here one C atom every 40 is excited).

We first consider an infinite straight carbon chains as a model. Two structures are considered here: cumulenes, where all C-C bondlengths are equal, resulting in a sequence of double bonds; and polyynes, where the C-C bondlengths alternate in a sequence of triple-single bonds. A useful parameter to distinguish between these two cases is the bond length alternation (BLA), defined as the difference between two subsequent bondlengths. For ideal infinitely long chains, polyynes (BLA >0) are more stable than cumulenes (BLA =0) [54,55]. The cumulene to polyyne switch is a Peierls-type distortion. We adopt for this analysis the interatomic distances from [54]. Figure 1a,b shows the band structure of cumulenes and polyynes, respectively. For better comparison, we present the band structure for cumulenes evaluated in a two-atom supercell (as necessary for polyynes). The corresponding bands near the Fermi level $E_{F}$ are degenerate at zone boundary for cumulenes, whereas the Peierls distortion opens a band gap making polyynes semiconducting. At the right of each band structure, the density of states (DOS) projected over C 2p atomic orbitals is reported. For both structures, the states around $E_{F}$ originate from p${}_{y}$ and p${}_{z}$ orbitals, i.e., those directed orthogonal to the chain (contributing equally by symmetry), whereas bonding and antibonding states with p${}_{x}$ character, i.e., aligned along the chain axis, are found much farther away from $E_{F}$.

Figure 1c reports the NEXAFS spectrum simulated in the half-core-hole approximation [45], for different photon polarizations, i.e., different directions of the photon electric field E: along the C chain axis (*x*) and perpendicular to it (*y* and *z*). We recall that these calculations are based on pseudopotential methods and as such the energies, not including the core-level binding energy, are referred to the average C 1s ionization potential $E_{\mathrm{thr}}^{\mathrm{avg}}$ (see Section 4). Both structures exhibit a peculiar dependence on the polarization direction (dichroic signal): in the lower-energy near-edge region (0–5 eV in the plot) only $\pi^{*}$ orbitals are available as final states for the electronic excitation. These originate from the C 2p${}_{y,z}$ states and, given the dipole selection rule with a C 1s initial state, they can only be excited by the photon field along *y* and *z* (blue curves). At higher energy, also states with $\sigma^{*}$ character (hybrid 2s and 2p${}_{x}$) can be excited with the photon field along the chain axis (red curves).

Differences between the two configurations appear for both polarizations. For E∥y,z, the metallic cumulene exhibits a smooth onset of the signal, whereas the one of the semiconducting polyyne is characterized by a strong peak associated to the Van Hove singularity of the conduction band bottom, shown in the PDOS of Figure 1b, enhanced by the interaction with the core hole. Another interesting difference is the energy position of the resonance in the E∥x spectra, associated to the $\sigma^{*}$ antibonding resonance. As such, this peak depends critically on the interatomic distance in molecules and, by inverting the relation, has been used for structural determination [56]. Here, in going from cumulene to polyyne, the relative position of this resonance is a result of the interplay between the elongation of one bond and the reduction of the other one, whose outcome is not easily predictable. According to our simulations, bond elongation is more effective and a shift of the resonance to lower energy can be appreciated.

**Figure 2:**
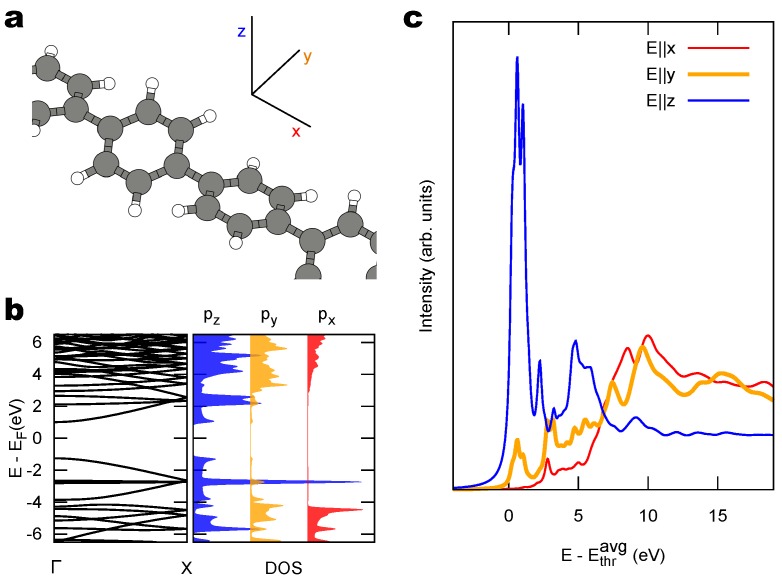
(**a**) Structural model of freestanding poly(p-phenylene) with alternating tilted phenyl rings and (**b**) the corresponding 1D band structure and PDOS. (**c**) Simulated NEXAFS spectrum for different photon polarizations (axes as in panel (**a**)).

To address the properties of sp${}^{1}$/sp${}^{2}$ structures we first analyze a pure sp${}^{2}$ polymer. Hence, as a prototype of NEXAFS dichroism in a sp${}^{2}$ system, we take poly(p-phenylene) (PPP), which is composed of repeated phenyl units and can be obtained by the polymerization of benzene [57]. Like in biphenyl and substituted biphenyls [58], the structure is stabilized by twisting adjancent phenyl units around the axis. We adopt here as a model structure a quasi-planar arrangement of the polymer, with two phenyl rings at $\pm 15^{{}^{\circ}}$ from a reference x,y plane, as determined by DFT: this model, depicted in Figure 2a, is both computationally more feasible and more representative of adsorbed molecules and polymers (discussed in the next paragraphs), than a helical arrangement having the twist angle of phenyl units increasing monotonously. Figure 2b reports the electronic band structure and the DOS projected on C 2p states. The PDOS shows the dominant π character of the frontier orbitals originating from atomic p${}_{z}$ states. Given the tilt of the aromatic rings, some contribution to those states is computed also for atomic orbitals with p${}_{y}$ symmetry, but truly σ states associated to p${}_{y}$ and p${}_{x}$ orbitals are only found farther away from the Fermi level.

Figure 2c shows the simulated NEXAFS spectrum of PPP, for photon polarizations along the polymer axis (*x*) and orthogonal to it (*y*, *z*). We are mostly interested to the lowest-energy features of these spectra, namely those corresponding to transitions to the lowest unoccupied states, LUMOs. These are the features typically used to determine tilt angles of aromatic systems deposited on a surface [36]. Despite the relative tilt angle between adjacent rings makes a distinction between π and σ states only approximate, the spectra bear the main features of planar sp${}^{2}$ aromatic hydrocarbons: the lowest unoccupied states have $\pi^{*}$ symmetry and are accessed mainly for E∥z; the spectrum with E∥x samples only states with $\sigma^{*}$ symmetry and yields no contribution in the lowest-energy resonances; the spectrum with E∥y mostly accesses final states with $\sigma^{*}$ symmetry but, because of tilted units, exhibits a non-vanishing intensity also in the lowest-energy region of $\pi^{*}$ states.

When we compare this sp${}^{2}$ polymer with the sp${}^{1}$ carbyne, the main difference is the following: the spectrum of the sp${}^{2}$ polymer (Figure 2c) is characterized by two in-plane directions, showing mainly σ character, plus one out-of-plane π-character direction; in contrast the sp${}^{1}$ carbyne spectrum (Figure 1c) exhibits one on-axis direction with σ character plus two off-axis directions with π character. This additional direction where π states can be accessed by NEXAFS becomes particularly interesting for the study of linear C structures on a surface.

### 2.2. Combined sp${}^{1}$/sp${}^{2}$ Polymers on a Metal Surface

**Figure 3:**
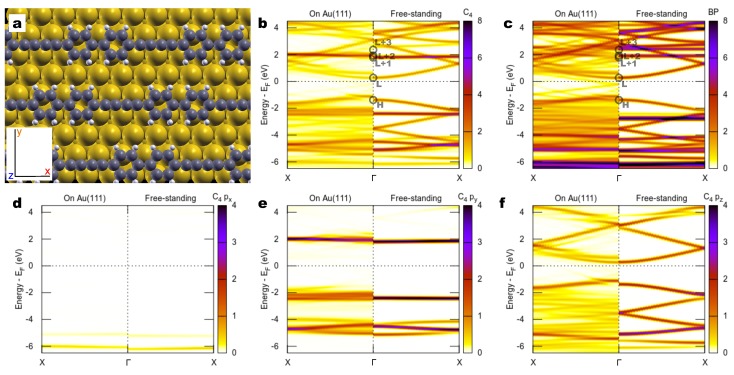
(**a**) Structural model of bBEBP/Au(111). (**b**,**c**) Electronic band structure projected on the C atoms belonging to the C${}_{4}$ section and the biphenyl one, respectively. Left and right panels report the calculations on Au(111) and free-standing, respectively. Darker colors indicate stronger localization over the C atoms, whereas white area denote the absence of electronic state projecting over the selected set of orbitals at that energy. The labeled circles mark the energy of highest occupied (H) and lowest unoccupied (L) electronic states, computed for the free-standing polymer, depicted in Figure 4. (**d**–**f**) The same band structure projected on the $p_{x}$, $p_{y}$, and $p_{z}$ orbitals of the C${}_{4}$ part.

We now move to the study of the actual system, namely a combined sp${}^{1}$/sp${}^{2}$ polymer recently synthesized [30], which can be considered a paradigmatic example of this class of combined sp${}^{1}$/sp${}^{2}$ materials. This polymer, grown on Au(111), consists of a chain where C${}_{4}$ linear units alternate with biphenyl groups (BP). We refer to the literature for the synthesis and STM characterization [30]. Here we simply recall that the polymer was synthesized by the polymerization of a molecular precursor (4,4${}^{\prime }$-di(bromoethynyl)-1,1${}^{\prime }$-biphenyl, bBEBP) through dehalogenative homocoupling, forming an intermediate organo-metallic compound (that we will return to below), and subsequent demetalation. Figure 3a shows a ball-stick model as determined by our DFT simulations performed at the adsorption site suggested by microscopy data [30]. The Methods section reports further details about the simulations.

**Figure 4:**
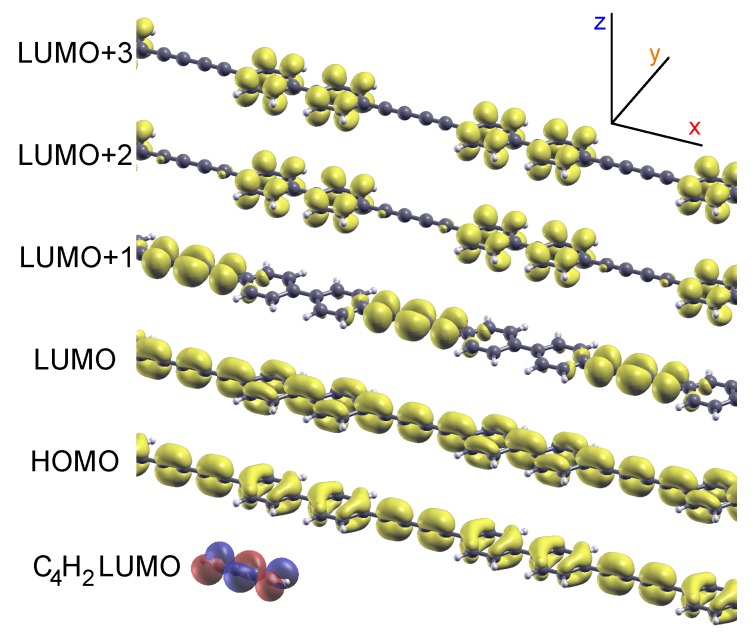
Isosurfaces corresponding to a squared-wavefunction amplitude of 0.01 Å${}^{-3}$, for the highest occupied (HOMO), the lowest unoccupied (LUMO), and a few more empty electronic states of the freestanding bBEBP polymer (in the geometry shown in Figure 3a, but without the Au surface atoms). For comparison to the LUMO + 1, the LUMO computed for C${}_{4}$H${}_{2}$ is shown at the bottom.

Before investigating the X-ray absorption spectra, it is useful to analyze the band structure of the polymer. To this purpose, Figure 3b–f reports the DOS of bBEBP/Au(111), projected on specific atomic orbitals of the polymer and resolved in wavevector *k* along the Γ-X direction. This *k*-resolved projected density of states (KPDOS) provides a color map of the relevant band structure, disentangling the polymer states from the substrate continuum of the Au surface. See, e.g., [59,60]. As a comparison, we compute the KPDOS also for free-standing bBEBP (i.e., once removing Au atoms from the full bBEBP/Au(111) structural model), as reported at the right-hand of each panel. Figure 3b,c focuses on the states belonging to the C${}_{4}$ and BP parts respectively. Consider first the freestanding case: one can see that several band states in the proximity of the Fermi level are hybrids involving both C${}_{4}$ and BP orbitals. For example, the lowest empty band, marked by “L” at the Γ point, is clearly visible in both b and c subpanels. Additionally a few states also appear to be due to C${}_{4}$ with no correspondence on the BP. In particular, a nearly flat band at ≈+2 eV in Figure 3b corresponds to a C${}_{4}$ state (labeled “L + 1”), whereas a band with similar dispersion and only slightly larger energy (“L + 2”, not easily resolved at the scale of the figure) is due to a state localized on the BP. This different localization of L + 1 and L + 2 states is better appreciated by looking at their wavefunction amplitude, computed at the Γ point for the free-standing polymer and reported in Figure 4. One sees that the highest occupied (H) and lowest unoccupied (L) bands are extended states involving p${}_{z}$ orbitals from both the C${}_{4}$ and the BP subsections. Contrarily, the L + 1 and L + 2 states are strongly localized to the C${}_{4}$ and to the BP units, respectively, with wavefunction amplitudes in close similarity to the ones of the corresponding molecular species. In particular, the L + 1 wavefunction amplitude shows striking similarity to that computed for the LUMO of gas-phase C${}_{4}$H${}_{2}$, also shown in Figure 4. The localized character of L + 1 and L + 2 states is also at the origin of their flat dispersion.

Further insight about the electronic states of the polymer is gained by inspecting Figure 3d–f, which reports the KPDOS on C${}_{4}$ states, here resolved into p${}_{x}$, p${}_{y}$, and p${}_{z}$ projections. The p${}_{x}$ contribution is practically negligible in the region reported, p${}_{z}$ states show appreciable dispersion because of coupling with ones with the same symmetry from BP, whereas the empty C${}_{4}$-localized states at ≈2 eV (L + 1) are shown to be of p${}_{y}$ symmetry as also deduced from Figure 4.

The results for bBEBP/Au(111) (left-hand subpanels) actually reproduce fairly closely the situation described so far for the free-standing case. The KPDOS reflects the hybridization of the polymer states with the Au(111) substrate bands, which in principle transforms the discrete levels into broad resonances, which practically appear as sequences of lower-intensity hybrid-interface bands in finite-size slab calculations [61,62]. Unsurprisingly, this phenomenon affects more the p${}_{z}$ states, which are directed toward the surface, than the p${}_{y}$ states, with consequences on the spectra as we will see below. The equivalent behavior of p${}_{y}$ and p${}_{z}$ states discussed in Section 2.1 for the free infinite carbyne is indeed lost for the finite C${}_{4}$ unit in the polymer due to its coupling to both the sp${}^{2}$ BP group and the substrate.

**Figure 5:**
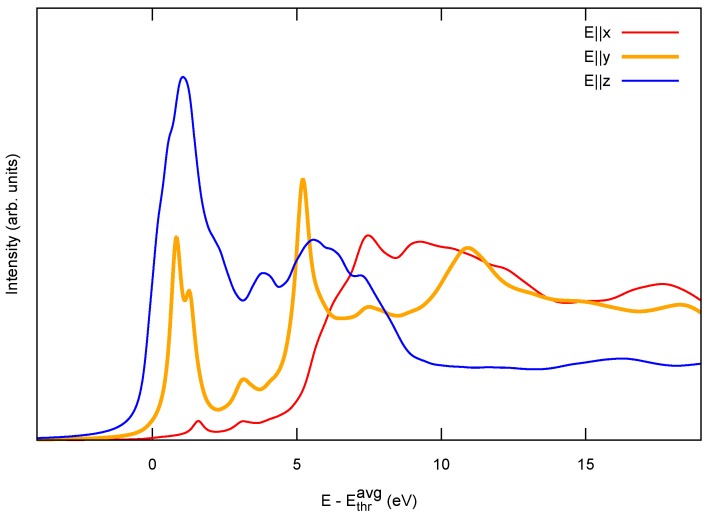
Simulated NEXAFS spectrum of bBEBP/Au(111). See Figure 3a for the definition of the x,y,z polarization axes.

We now come to the polarization-resolved NEXAFS spectrum of bBEBP/Au(111), reported in Figure 5. Compared to the spectrum of Figure 2c or to the NEXAFS spectrum of a typical aromatic system that are characterized by low-energy resonances only accessible with the electric field perpendicular to the molecular plane, here a sizable spectral feature is also predicted in the in-plane E∥y-polarized spectrum. This peak is due to electronic transitions to the states with p${}_{y}$ character. It exhibits a spectral shape that differs significantly from that with E∥z; the occurrence of a different lineshape is at variance with the case of PPP, Figure 2c, where a residual intensity in the *y* direction, having a similar profile as in the *z* direction, was only due to the tilted structure. In actual experiments, domains with different orientations of the polymers are found. As a result, measurements would average the *x* and *y* line shapes still resulting on a characteristic peak for in-plane polarization at a low energy typical of π states, where pure sp${}^{2}$ systems only exhibit out-of-plane character instead.

## 3. Discussion

### 3.1. Identification of sp${}^{1}$ C Chains in NEXAFS Spectra

**Figure 6:**
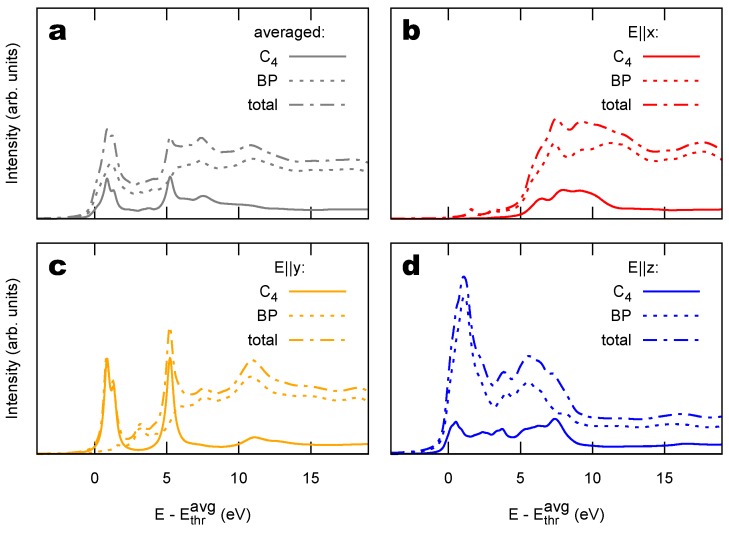
Decomposition of the NEXAFS spectrum of bBEBP/Au(111) into contributions by the sp${}^{1}$ chain (C${}_{4}$) and the sp${}^{2}$ biphenyl group (BP). (**a**) Spectrum averaged over all polarizations. (**b**) In-plane electric field along the polymer axis *x*, and (**c**) orthogonal to it, *y*; (**d**) out-of-plane *z*-directed electric field (see Figure 3a for the definition of x,y,z axes).

The NEXAFS spectra of bBEBP/Au(111) can be decomposed into contributions originating from the C${}_{4}$ and BP parts, as we do in Figure 6 for the spectrum averaged over the polarizations (panel a), and for the polarized spectra (panels b–d). Individual contributions by the various possible initial core-hole sites are reported in the Supplementary Materials, Figure S1. The BP C atoms are three times more numerous than the C atoms belonging to C${}_{4}$, and therefore dominates the spectra, with the notable exception of the E∥y polarized one shown in panel c. There one sees that the lowest-energy NEXAFS peak is indeed given by states belonging to the C${}_{4}$ unit and strictly due to the sp${}^{1}$ hybridization of the corresponding orbitals. One also observes that these spectral features are more resolved than the ones in panel d because of smaller hybridization with the substrate orbitals of the in-plane p${}_{y}$ states than of out-of-plane p${}_{z}$ ones, consistently with the ground-state band structure of Figure 3e–f. This larger spectral broadening facilitates the identification of out-of-plane contributions. Overall, the occurrence of characteristic NEXAFS peaks measured with in-plane polarization, also at low energy, could be used in experiments to fingerprint the presence of sp${}^{1}$hybridization in the C system.

### 3.2. Other Structural Models

**Figure 7:**
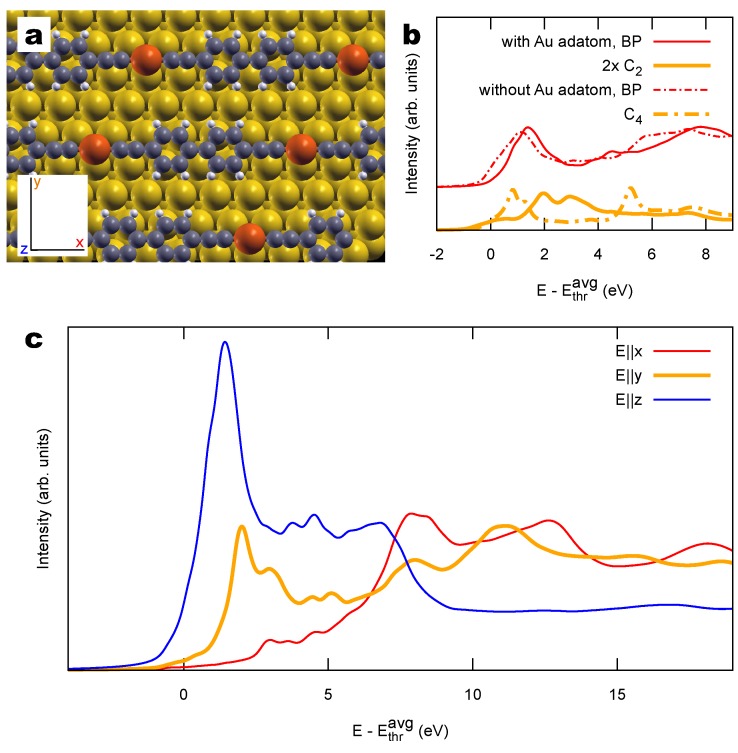
(**a**) Structural model of bBEBP/Au(111) embedding a Au adatom (red) in between two organic units. (**b**) Simulated polarization-averaged NEXAFS spectrum (solid) compared to the one of Figure 6a (without the Au adatom, dashed). (**c**) Polarization-resolved NEXAFS spectrum; see panel (**a**) for the definition of the x,y,z polarization axes.

To discuss the generality of the results presented for bBEBP/Au(111), we consider two different structural models. First we take the case of an intermediate stage in the formation of bBEBP/Au(111). This results from the dehalogenative homocoupling of the precursors, leaving to an organo-metallic compound where Au adatoms are embedded into polymer units. A subsequent thermal annealing would then remove the Au adatom from the intermediate structure and generate the bBEBP/Au(111) analyzed in the previous section [30]. To model the intermediate compound we consider the insertion of one Au adatom in our (5×3) supercell, resulting in a longer (6×3) one, and optimize the structure as discussed in Section 4. We consider the same alignment of the polymer relative to the substrate crystallographic directions as for the final product (cf. Figure 3a) and obtain a flat-lying and slightly elongated polymer depicted in Figure 7a. The presence of the Au atom influences only minimally the contribution by the BP part to the NEXAFS spectra, as shown by the averaged spectra in Figure 7b. Instead, the sp${}^{1}$ section, now only consisting of extremely short C${}_{2}$ fragments, gives a strongly modified spectrum. In particular, as can be seen by inspecting the full polarized spectrum shown in Figure 7c, the peaks seen in *y*-polarization are now at slightly larger energy than those seen in *z*-polarization, at variance with the result of the final polymer seen in Figure 5 where an opposite energy alignment is observed. This can be explained by the widening of the HOMO-LUMO gap as the linear chain shortens, bringing the LUMO at higher energy. Nevertheless, the full spectrum shown in Figure 7c still reports a significant polarization dependence and emergence of a low-energy signal for in-plane directions. Additional detail about these spectra is provided in the Supplementary Materials, Figures S2 and S3.

**Figure 8:**
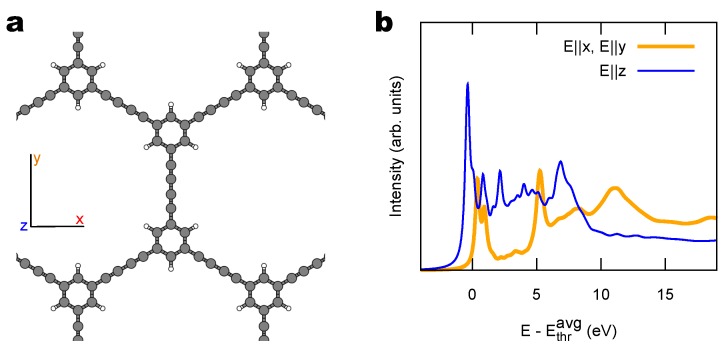
(**a**) Structural model of 2D network formed by de-bromination of tBEP/Au(111) [30] (here simulated as if it were freestanding). (**b**) Simulated NEXAFS spectrum for in-plane (x,y) and out-of-plane (*z*) photon polarizations.

While holding on to the need of stabilizing sp${}^{1}$ chains by aromatic units anchoring to the substrate, the investigation can be extended to non-linear polymeric arrangements of the monomers as, e.g., obtained with carbon-based scaffolds [63]. As an example, consider the 2D network formed by the de-Bromination and subsequent de-metalation of 1,3,5-tris(bromoethynyl)benzene (tBEP) precursors deposited on Au(111) [30]. This sequence of reactions forms a honeycomb lattice where phenyl rings are connected by C${}_{4}$ segments arranged at 120${}^{{}^{\circ}}$ from each other, as illustrated in Figure 8a. Given the relatively limited role of the substrate in the electronic structure of the polymer discussed in Section 2.2, and the necessarily larger unit cell, here we only report simulations of the free-standing case. The structure is optimized by minimizing the DFT total energy. Figure 8b shows the computed NEXAFS spectrum for this system. Very much as in the linear bBEBP polymer where the LUMO + 1 states of Figure 4 are highly localized on the C${}_{4}$ units and consequently appear as flat bands in Figure 3e, also this 2D compound has similar C${}_{4}$-localized empty states derived from in-plane C 2p orbitals, which produce a similar polarization-dependent NEXAFS signal, with low-energy resonances occurring even for in-plane polarization. Few differences are worth pointing out for this case: here the *x* and *y* directions are equivalent due to hexagonal symmetry; the first peak in the *z* spectrum is narrower than in bBEBP/Au(111) because of the simpler aromatic ligand (as well as due to the model neglecting substrate hybridization); the relative intensity of the in-plane features increases, since the number of sp${}^{1}$ atoms is now the same as that of sp${}^{2}$ ones. Especially this last aspect is interesting as it would make sp${}^{1}$ easier to detect in experiments.

## 4. Materials and Methods

### 4.1. Theoretical Framework

Our first-principle calculations are based on the DFT [41,42]. For the exchange and correlation functional we take the generalized gradient approximation (GGA) by Perdew, Burke, and Ernzerhof (PBE) [64,65]. We model the systems by periodically repeated unit cells/supercells. We represent the electronic wavefunctions on a plane-wave basis, using pseudopotentials as implemented in the Quantum-ESPRESSO distribution [66,67] to represent the core electrons. Pseudopotentials used here are the same as in previous works [52,60,68].

We computed the NEXAFS spectra within the half-core-hole approximation [45] to Slater’s transition-state theory [44]. Here, the excitation is modeled by evaluating the electronic structure for the outer electrons (final states) in the presence of a fractional core occupancy (core configuration: 1s${}^{1.5}$) at the excited atom (initial state). The numerically heavy summation over the final states, weighted by the transition matrix elements, is replaced by a Lanczos approach, as implemented in the XSpectra package of Quantum-ESPRESSO [69]. A limitation of this approach lies in its reliance on final-state energies at the DFT-PBE level: given the overestimated electron affinities, one computes bound states below the vacuum level that in reality should be resonances in the unbound continuum. This artifact leads to spectral features that are overly enhanced in the simulation in the intermediate energy range between bound and unbound states [52]. We recall that, since calculations are based on the pseudopotential approach, transition energies and core-level binding energies are only given up to a constant. Following [52], we determine relative binding energies (core-level shifts) by the full-core-hole (FCH, core configuration: 1s${}^{1.0}$) approach [70], and use those energies to align the spectral contributions of different initial states. As a result, spectra are referenced to the average XPS energy $E_{\mathrm{thr}}^{\mathrm{avg}}$, as measured from the Fermi level [50]. It may also help to note that, in cases where all C atoms in the system are equivalent, as the sp${}^{1}$ chains of Section 2.1, the resulting spectra are simply aligned to the Fermi level.

### 4.2. Numerical Protocol

Detailed structural optimizations and energetics are also accessible within the same DFT framework but go beyond the scope of the current work, which is instead focused on highlighting the X-ray absorption spectral dependence on the atomistic arrangement in a broader perspective. For these reasons, structures have been taken from the literature when available. In particular, we take the bond distances for carbynes of polyynes and cumulenes from the literature [54] with values of 1.207/1.379 Å and of 1.282 Å for triple/single and double bonds, respectively. We also remark that optimized BLA and relative energy differences for carbynes strongly depend on the theoretical method [71].

The structures for bBEBP/Au(111) are derived from our previous investigation [72] of the same system, using a localized basis set as implemented in the SIESTA code [73]. The polymer is aligned along a close-packed direction of the substrate, in agreement with the available STM images and proposed models [30]. For the present simulations we have adopted a (5×3) surface unit cell and ignored the likely presence of residual Br atoms as well as other contaminants and defects. We performed the atomic relaxation restricted to the polymer (force cutoff equal to 0.06 eV/Å). The substrate atoms were kept fixed to the coordinates of the relaxed clean surface, which was represented by 6 gold layers, with an interposed vacuum of 28 Å.

The molecule-surface Van der Waals interaction was introduced via a dispersion potential of the Grimme type. The relaxed geometry, including the equilibrium adsorption distance, reproduce the results reported in literature [30]. We compared the DOS computed by SIESTA and Quantum-ESPRESSO and found a very good agreement between the electronic properties of the system obtained in the two approaches.

For what concerns the organometallic chain of Section 3.2, the structural model proposed by Sun et al. introduced a misalignment angle of the polymer relative to the close-packed Au(111) direction. Such a tilt is not commented by the authors of that work [30] nor, in our opinion, evidently supported by the experimental STM images presented therein which report crystallographic directions together with the observed polymer lines. Differently, in our theoretical model we have chosen to maintain the alignment to the close-packed direction and a (6×3) unit cell, longer by one Au-Au distance compared to the case of the bBEBP/Au(111) of Section 2.2, to accommodate the extra Au adatom along the polymer chain. For that adatom, we have chosen the face-centered cubic (fcc) hollow adsorption site. We recall that in actual experiments several different configurations and orientations are observed, as well as different adsorption sites for the Au adatom (e.g., hexagonal close packed, hcp hollow), but the essential aspects are expected to be mildly dependent on the specific conditions as supported by the overall similarity of the band structures of adsorbed and freestanding polymers seen Figure 3. The relaxed organometallic chain is slightly bent, with the Au atom lying slightly below the aromatic part at a distance from the surface of 2.55 Å. We also considered different spacing between the organometallic chain by taking larger (6×5) periodicity, and verified that the local molecule-surface interaction and the electronic properties of the chain are weakly affected by the spacing between the chains.

We have optimized the structures of free-standing PPP (with two oppositely-tilted phenyl units per supercell), and tBEP within DFT-PBE with Quantum-ESPRESSO.

We have simulated the NEXAFS spectra of 1D carbynes in supercells where one C atom out of 40 is excited. Smaller supercells (with 20 atoms) amplify the wiggles that are seen in the spectra (Figure 1c) but already reproduce the main findings as shown in the Supplementary Materials, Figure S4. We have taken 4 *k*-points along the chain direction, sufficient to produce fully convergent spectra. The NEXAFS spectrum of the PPP polymer was determined for a supercell including 48 C atoms, also with a 4-*k*-point mesh.

For bBEBP/Au(111), we consider a (5×3) substrate periodicity, which includes one unit cell of the polymer. The use of repeated supercells was in this case not necessary, given the screening by the Au substrate, and we verified that the results were accurately reproduced with a doubled supercell. We sampled the *k* points on a (6×5) grid of the surface Brillouin zone.

Similarly, we computed the electronic structure of metal-intercalated bBEBP/Au(111) adopting the (6×3) substrate periodicity which includes one unit cell of the polymer, and with an equivalent (5×5)*k*-point sampling. Finally, the free-standing hexagonal lattice of tBEP is described by its 30-atom unit cell with a (6×6)*k*-point sampling. We checked that the NEXAFS spectrum converges already at much lower plane-waves cutoffs than those necessary for total-energy and geometry calculations, so we have taken reduced values (27 Ry) for the reported results.

## 5. Conclusions

We simulated by first principles the electronic properties and NEXAFS spectra of C systems with pure and with combined sp${}^{1}$ and sp${}^{2}$ character. Given their dimensionality, linear sp${}^{1}$ chains are characterized by off-axis C 2p states along two directions (say *y* and *z*), at variance with sp${}^{2}$ systems where only one off-plane direction is present (*z*). The contributions of such off-axis/off-plane typically characterize the low-energy part of the NEXAFS spectrum, so that sp${}^{1}$ systems are predicted to exhibit intense X-ray absorption at the onset for two polarizations of the photon electric field. In combined systems, or for linear C chains as grown at a surface, p${}_{z}$ states would couple to the sp${}^{2}$ part and/or to the substrate, thus remaining largely obscured. Our main prediction is that p${}_{y}$ states will instead contribute a characteristic low-energy peak to adsorption of X-rays polarized parallel to the surface plane. We suggest that this low-energy feature could be used to fingerprint the presence of sp${}^{1}$ hybridization by polarized NEXAFS measurements.

## Supplementary Materials

The following are available online at http://www.mdpi.com/1996-1944/11/12/2556/s1, Figure S1: (a) Structural model and inequivalent carbon atoms in bBEBP/Au(111). (b) Decomposition of the NEXAFS spectrum of bBEBP/Au(111) into the individual initial-state contributions, as numbered in panel (a), Figure S2: (a) Structural model and inequivalent carbon atoms in bBEBP/Au(111) embedding a Au adatom (red) in between two organic units. (b) Decomposition of the corresponding NEXAFS spectrum into the individual initial-state contributions, as numbered in panel (a), Figure S3: Decomposition of the NEXAFS spectrum of bBEBP/Au(111) embedding a Au adatom on contributions by the sp${}^{2}$ biphenyl part (BP) and the sp${}^{1}$ chains (C${}_{2}$). (a) Spectrum averaged over the polarizations. (b) In-plane electric field along the polymer axis *x* and (c) orthogonal to it, *y*; (d) out-of-plane electric field, *z*. See Figure S2a for the definition of x,y,z axes, Figure S4: NEXAFS spectrum of cumulenes (left) and polyynes (right) computed with a 20-atom and 40-atom simulation supercell. One atom per cell is excited. Polarization directions are taken with *x* along the chain axis and *y*, *z* orthogonal to it.

## Abbreviations

The following abbreviations are used in this manuscript:

1D	one-dimensional
2D	two-dimensional
AFM	atomic force microscopy
bBEBP	4,4${}^{\prime }$-di(bromoethynyl)-1,1${}^{\prime }$-biphenyl
BLA	bond length alternation
BP	biphenyl
DOS	density of states
DFT	density functional theory
FCC	face-centered cubic
HCP	hexagonal close packed
KPDOS	*k*-resolved projected density of states
NEXAFS	near-edge X-ray absorption fine structure
PBE	Perdew, Burke, and Ernzerhof
PDOS	projected density of states
PPP	poly(p-phenylene)
STM	scanning tunneling microscopy
tBEB	1,3,5-tris(bromoethynyl)benzene
UHV	ultra-high vacuum
XPS	X-ray photoemission spectroscopy
