# Supplementary material for: Fingerprints of sp1 Hybridized C in the Near-Edge X-ray Absorption Spectra of Surface-Grown Materials
Source: Materials (Basel). 2018 Dec 15;11(12):2556. doi: 10.3390/ma11122556 (PMC6315668; doi:10.3390/ma11122556)
Supplement: Supplementary file 1 [file materials-11-02556-s001.pdf]

Article

Fingerprints of sp^1 Hybridized C in the Near-Edge X-Ray Absorption Spectra of Surface-Grown Materials Supplementary Materials

Guido Fratesi[1,*], Simona Achilli[1], Nicola Manini[1], Giovanni Onida[1], Anu Baby[2], Abhilash Ravikumar[2], Aldo Ugolotti[2], Gian Paolo Brivio[2], Alberto Milani[3] and Carlo Spartaco Casari[3]

[1] ETSF and Dipartimento di Fisica, Università degli Studi di Milano, Via Celoria, 16, 20133 Milano, Italy
[2] Dipartimento di Scienza dei Materiali, Università di Milano-Bicocca, Via Cozzi, 55, 20125 Milano, Italy
[3] Department of Energy, Politecnico di Milano via Ponzio 34/3, I-20133 Milano, Italy
* Correspondence: guido.fratesi@unimi.it; Tel.: +39-02-503-17348

Version November 22, 2018 submitted to Materials; Typeset by LaTeX using class file mdpi.cls

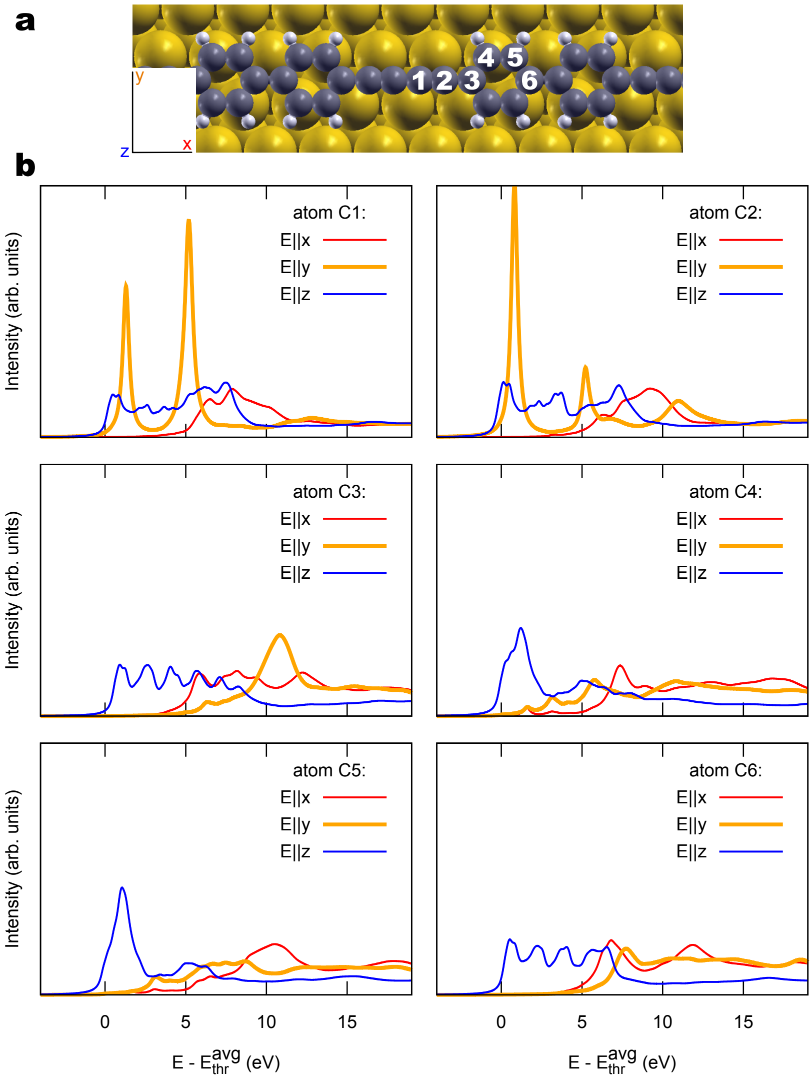

Figure S1. (a) Structural model and inequivalent carbon atoms in bBEBP/Au(111). (b) Decomposition of the NEXAFS spectrum of bBEBP/Au(111) into the individual initial-state contributions, as numbered in panel (a).

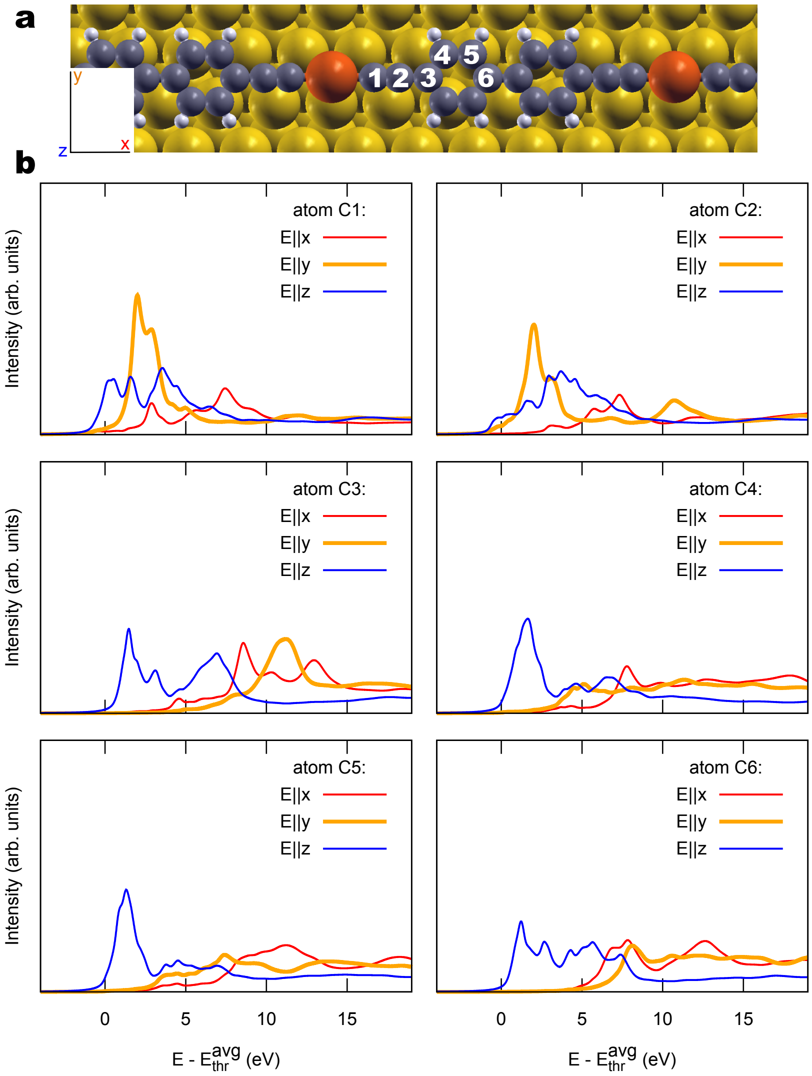

Figure S2. (a) Structural model and inequivalent carbon atoms in bBEBP/Au(111) embedding a Au adatom (red) in between two organic units. (b) Decomposition of the corresponding NEXAFS spectrum into the individual initial-state contributions, as numbered in panel (a).

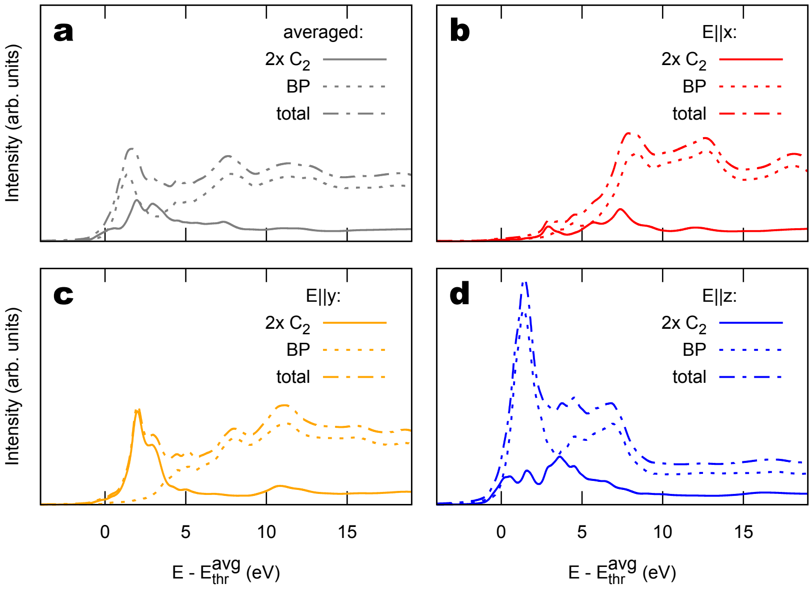

Figure S3. Decomposition of the NEXAFS spectrum of bBEBP/Au(111) embedding a Au adatom on contributions by the sp^2 biphenyl part (BP) and the sp^1 chains (C_2). (**a**) Spectrum averaged over the polarizations. (**b**) In-plane electric field along the polymer axis x and (**c**) orthogonal to it, y; (**d**) out-of-plane electric field, z. See Figure S2a for the definition of x, y, z axes.

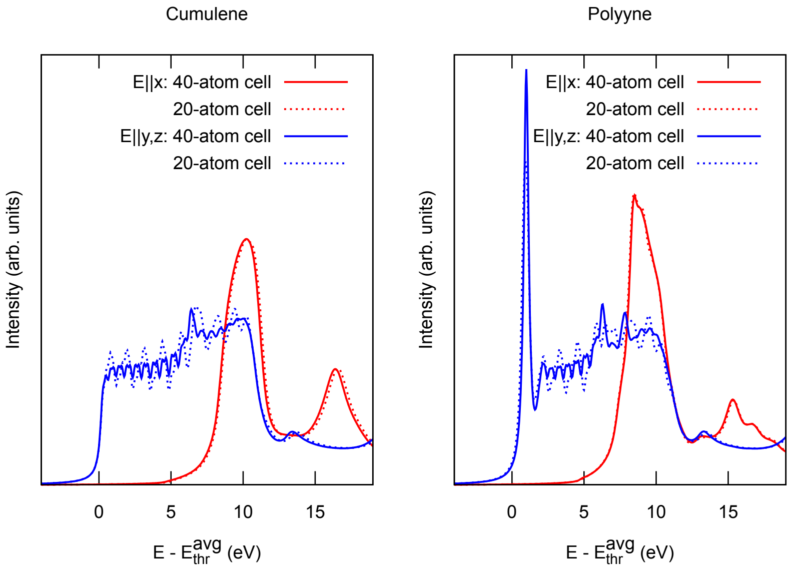

Figure S4. NEXAFS spectrum of cumulenes (left) and polyynes (right) computed with a 20-atom and 40-atom simulation supercell. One atom per cell is excited. Polarization directions are taken with x along the chain axis and y, z orthogonal to it.

1 © 2018 by the authors. Submitted to *Materials* for possible open access publication under the terms and
2 conditions of the Creative Commons Attribution license (http://creativecommons.org/licenses/by/4.0/)
